# Comparison of SAPS 3 performance in patients with and without solid tumor admitted to an intensive care unit in Brazil: a retrospective cohort study

**DOI:** 10.5935/0103-507X.20200089

**Published:** 2020

**Authors:** Leandro Utino Taniguchi, Ellen Maria Pires Siqueira

**Affiliations:** 1 Hospital Sírio-Libanês - São Paulo (SP), Brazil.; 2 Emergency Medicine Discipline, Hospital das Clínicas, Faculdade de Medicina, Universidade de São Paulo - São Paulo (SP), Brazil.; 3 Brazilian Research in Intensive Care Network (BRICNet) - São Paulo (SP), Brazil.

**Keywords:** Simplified acute physiology score, Prognostic, Neoplasms, Intensive care units, Escala fisiológica aguda simplificada, Prognóstico, Neoplasias, Unidades de terapia intensiva

## Abstract

**Objective:**

To compare the performance of the Simplified Acute Physiology Score 3 (SAPS 3) in patients with and without solid cancer who were admitted to the intensive care unit of a comprehensive oncological hospital in Brazil.

**Methods:**

We performed a retrospective cohort analysis of our administrative database of the first admission of adult patients to the intensive care unit from 2012 to 2016. The patients were categorized according to the presence of solid cancer. We evaluated discrimination using the area under the Receiver Operating Characteristic curve (AUROC) and calibration using the calibration belt approach.

**Results:**

We included 7,254 patients (41.5% had cancer, and 12.1% died during hospitalization). Oncological patients had higher hospital mortality than nononcological patients (14.1% *versus* 10.6%, respectively; p < 0.001). SAPS 3 discrimination was better for oncological patients (AUROC = 0.85) than for nononcological patients (AUROC = 0.79) (p < 0.001). After we applied the calibration belt in oncological patients, the SAPS 3 matched the average observed rates with a confidence level of 95%. In nononcological patients, the SAPS 3 overestimated mortality in those with a low-middle risk. Calibration was affected by the time period only for nononcological patients.

**Conclusion:**

SAPS 3 performed differently between oncological and nononcological patients in our single-center cohort, and variation over time (mainly calibration) was observed. This finding should be taken into account when evaluating severity-of-illness score performance.

## Abstract (pt)

**Objetivo:**

Comparar o desempenho do *Simplified Acute Physiology Score 3* (SAPS 3) em pacientes com e sem tumor sólido admitidos à unidade de terapia intensiva de um hospital oncológico.

**Métodos:**

Realizamos a análise de uma coorte retrospectiva em nossa base de dados administrativa da primeira admissão de pacientes adultos à unidade de terapia intensiva entre 2012 e 2016. Os pacientes foram classificados segundo a presença de tumor sólido. Avaliamos a discriminação utilizando a área sob a curva Características de Operação do Receptor (ASC COR) e a calibração com uso da abordagem com faixa de calibração.

**Resultados:**

Incluímos 7.254 pacientes (41,5% tinham câncer e 12,1% morreram durante a hospitalização). Os pacientes oncológicos tiveram maior mortalidade hospitalar do que os não oncológicos (respectivamente, 14,1% e 10,6%; p < 0,001). A discriminação do SAPS 3 foi melhor para os pacientes oncológicos (ASC COR = 0,85) do que para os não oncológicos (ASC COR = 0,79) (p < 0,001). Após aplicar a abordagem com faixa de calibração para pacientes oncológicos, o SAPS 3 atingiu as taxas médias observadas com intervalo de confiança de 95%. Em pacientes não oncológicos, o SAPS 3 superestimou a mortalidade nos pacientes com risco baixo a moderado. A calibração foi afetada pelo tempo apenas nos casos de pacientes não oncológicos.

**Conclusão:**

O SAPS 3 teve desempenho distinto para pacientes oncológicos e não oncológicos em nossa coorte de um único centro, observando-se variação (principalmente da calibração) ao longo do tempo. Esses achados devem ser levados em consideração ao avaliar o desempenho de escore de doença grave.

## INTRODUCTION

Severity-of-illness scores are used in intensive care unit (ICU) settings worldwide for performance evaluation and monitoring, quality improvement and benchmarking.^(1-3)^ Since their first description in the 1980s, many prognostic models have been developed. However, among them, the Simplified Acute Physiology Score 3 (SAPS 3) is the only one developed from a multinational cohort (16,784 patients from 35 countries).^(4,5)^ In Brazil, Moralez et al. recently demonstrated that SAPS 3 (standard equation) remains the most accurate prognostic model.^(6)^ However, the performance of severity-of-illness scores might be different in some institutions due to the case-mix and representativeness of subgroups, such as oncological patients.

Because intensivists are increasingly managing oncological patients,^(7)^ studies evaluating the performance of these prognostic models in this subgroup of critically ill patients are welcomed. Although several studies have been published concerning this topic,^(8-10)^ they were published almost ten years ago, and a well-known phenomenon that compromises the performance of these prognostic models is deterioration over time (mainly in calibration) as previously demonstrated.^(8,11)^ None of these studies compared the model performance in oncological *versus* nononcological patients.

Therefore, our objectives in the present study were to evaluate the performance of SAPS 3 in patients with cancer admitted to a Brazilian ICU, compare the SAPS 3 performance of patients with and without cancer and to study time trends in SAPS 3 performance.

## METHODS

This was a retrospective cohort study of all consecutive patients admitted to our medical-surgical ICU (a 30-bed unit at *Hospital Sírio-Libanês*, a private tertiary hospital with a dedicated oncology unit, located in São Paulo, Brazil) and was approved by the local ethics committee. The detailed description of our unit was previously published and did not change during the study period.^(12)^ The exclusion criteria were age younger than 18 years and pregnancy. If the patients had more than one admission during the inclusion period, only the first admission was included. Some of the patients included in this study were also included in a previous analysis of our group regarding ICU readmission (1,702 patients).^(12)^

Our analysis used administrative data that were prospectively collected in a cloud-based software database (Sistema Epimed™) by trained nurses.^(13)^ The study period was from January 1st, 2012 to July 31st, 2016. The oncological condition was defined as any patient admitted with an active solid cancer (current curative or palliative chemotherapy, radiotherapy, immunotherapy or surgery) in the last 12 months. Hematological patients were excluded because they usually have distinct characteristics compared with patients with solid tumors (e.g., a higher burden of active disease; required oncological treatment during ICU stay; prolonged duration of neutropenia; a higher intensity of immunosuppression; concomitant bone marrow transplant; and a higher incidence of specific complications, such as invasive mold fungal infections and cytomegalovirus infection).

The data recorded included age, sex, date of ICU admission, SAPS 3,^(4,5)^ referring facility, admission diagnosis, surgical procedures before admission, Charlson index for comorbidities,^(14)^ resource utilization during ICU stay (mechanical ventilation, vasoactive drugs or renal replacement therapy), oncological status (locoregional or metastatic) and hospital mortality. The SAPS 3 was calculated using data from the ICU admission. As recommended, missing values were coded as “normal” for each variable.^(6)^

### Data analysis

The primary outcome was hospital mortality. Quantitative parametric data were presented as means ± standard deviation (SD), nonparametric data were presented as medians (25% - 75% interquartile range - IQR), and categorical variables were presented as percentages. Categorical variables were compared using chi-squared test.

The primary outcome was hospital mortality. The estimated mortality rate was calculated using the standard equation for the SAPS 3. SAPS 3 discrimination was evaluated using the area under the Receiver Operating Characteristic curve (AUROC). Comparisons between AUROCs were performed using the Delong method.^(15)^ Calibration was assessed using the calibration belt method as described by the GiViTI group.^(16)^ This method applies a generalized polynomial logistic function between the outcome and logit transformation of the estimated predicted probability, with the respective 95% and 80% confidence interval (CI) boundaries. A statistically significant deviation from the bisector (the line of perfect calibration) occurs when the 95%CI boundaries of the calibration belt do not include the bisector.^(16)^ Standardized mortality rates (SMRs) with 95%CIs were calculated by dividing the observed by the predicted mortality rates. The Brier score is an overall performance measure that was calculated using a standard formula.^(17)^ To study time trends in the SAPS 3 performance, we split the cohort into two subgroups by the ICU admission date (October 1st, 2014 to create two subgroups with similar sizes).

The data were analyzed using IBM SPSS Statistics, Version 21 and R (http://www.r-project.org). All the statistics were two-tailed, and a p value < 0.05 was considered statistically significant.

## RESULTS

During the study period, 8,345 ICU admissions occurred. After excluding 846 readmissions and 945 hematological patients, 7,390 patients remained. However, 136 patients (1.8%) did not have a hospital discharge status in our database. Therefore, our final study cohort comprised 7,254 patients (41.5% had cancer). Oncological patients were younger, had a lower SAPS 3, were admitted more frequently after elective surgery, and had higher hospital mortality than nononcological patients (14.1% *versus* 10.6%, respectively; p < 0.001; Table 1).

**Table 1:** General characteristics of the included patients

	All patients	Oncological	Nononcological	p value
n****	7.254	3.008	4.246	
Age (years)	66.2 ± 18.4	62.6 ± 16.6	68.7 ± 19.2	< 0.001
Male	3911 (53.9)	1711 (56.9)	2200 (51.8)	< 0.001
SAPS 3	41 (32 - 51)	38 (30 - 51)	42 (33 - 51)	< 0.001
Admission type				< 0.001
Medical	3.744 (51.6)	1.014 (33.7)	2.730 (64.3)	
Emergency surgery	640 (8.8)	176 (5.9)	464 (10.9)	
Elective surgery	2.867 (39.5)	1.817 (60.4)	1.050 (24.7)	
Length of hospital stay before ICU admission (days)	1 (0 - 2)	1 (0 - 3)	1 (0 - 2)	< 0.001
Charlson comorbidity index	2 (0 - 3)	2 (2 - 6)	0 (0 - 1)	< 0.001
Performance status*				< 0.001
Independent	3.698 (80.3)	1.652 (89.2)	2.046 (74.3)	
Assistance required	547 (11.9)	128 (6.9)	419 (15.2)	
Restricted/bedridden	362 (7.9)	73 (3.9)	289 (10.5)	
Most common primary tumor site				
Colon	---	538 (17.9)	---	
Central nervous system	---	471 (15.7)	---	
Pancreas	---	265 (8.8)	---	
Lung	---	260 (8.6)	---	
Type of cancer				
Locoregional	---	2.147 (71.4)	---	
Metastatic	---	861 (28.6)	---	
Main ICU admission diagnosis				
Gastrointestinal	1.575 (21.7)	975 (32.4)	520 (12.2)	
Neurological	1.385 (19.1)	662 (22.0)	723 (17.0)	
Infection	1.368 (18.9)	393 (13.1)	975 (23.0)	
Mechanical ventilation during ICU stay	1.398 (19.3)	573 (19)	825 (19.5)	0.67
Vasopressors during ICU stay	2.054 (28.3)	874 (29.1)	1.180 (27.8)	0.25
Renal replacement therapy during ICU stay	368 (5.1)	97 (3.2)	271 (6.4)	< 0.001
Hospital mortality	876 (12.1)	424 (14.1)	452 (10.6)	< 0.001

SAPS 3 - Simplified Acute Physiology Score 3; ICU - intensive care unit.

* Among 4,607 patients for whom data could be ascertained. Results expressed as means ± standard deviation, n (%) or medians (25% - 75% interquartile range).

Using the standard SAPS 3 predictive equation, the overall SMR was 0.93 (95%CI 0.88 - 0.98), with a Brier score of 0.088 and an AUROC of 0.82 (95%CI 0.81-0.83). Calibration belt analysis showed that SAPS 3 tended to overestimate mortality in our entire cohort of patients (Table 2).

**Table 2:** Comparison of SAPS 3 performance in all patients and subgroups of oncological and nononcological patients

	SMR (95%CI)	Discrimination AUROC (95%CI)	Calibration*		Precision
			Over the bisector 95%CI	Under the bisector 95%CI	Brier score
All patients	0.93 (0.88 - 0.98)	0.82 (0.81 - 0.83)	Never	0.18 - 0.80	0.088
Oncological patients	1.09 (1.01 - 1.17)	0.85 (0.83 - 0.86)	Never	Never	0.096
Nononcological patients	0.82 (0.75 - 0.89)	0.79 (0.78 - 0.81)	Never	0.08 - 0.43	0.082

SMR - standardized mortality rate; 95%CI - 95% confidence interval; AUROC - area under the Receiver Operating Characteristic curve.

* Calibration described as the bisector deviation intervals using the calibration belt method.

### Comparison of the SAPS 3 performance in oncological patients and nononcological patients

**Figure 1:**
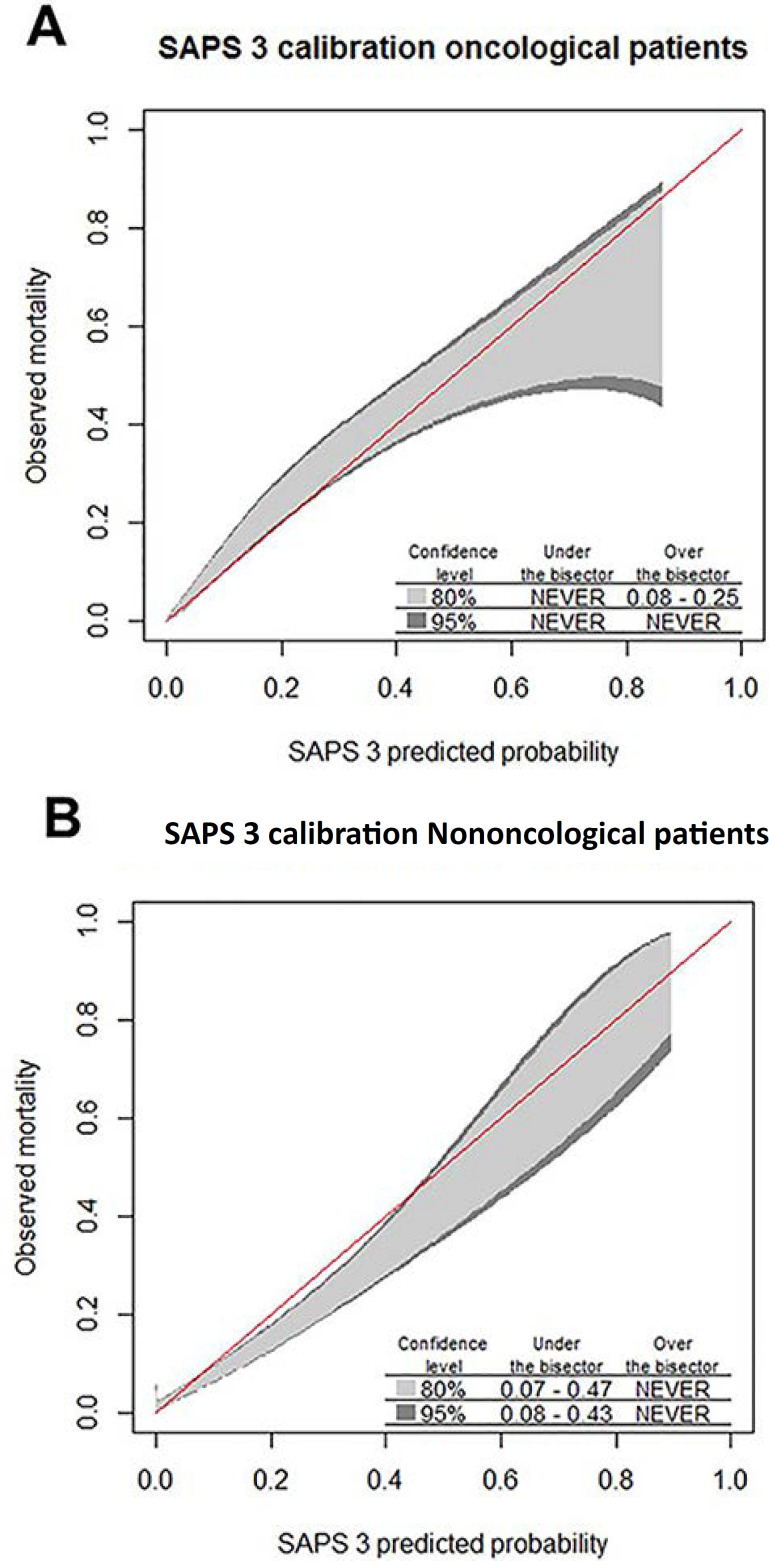
Calibration belt for Simplified Acute Physiology Score 3 in oncological (A) and nononcological patients (B), described as bisector deviation intervals. The predicted mortality intervals at which the calibration belt significantly deviates from the bisector and the 80% and 95% confidence levels are described in the lower right region of the plots. SAPS 3 - Simplified Acute Physiology Score 3.

The discrimination of SAPS 3 was higher for oncological patients than for nononcological patients (p < 0.001; Table 2). Calibration belt analysis demonstrated that, in oncological patients, no miscalibration was observed. However, in nononcological patients, SAPS 3 overestimated mortality in those with low-middle predicted risk (Figure 1).

### Time trend evaluation

**Figure 2:**
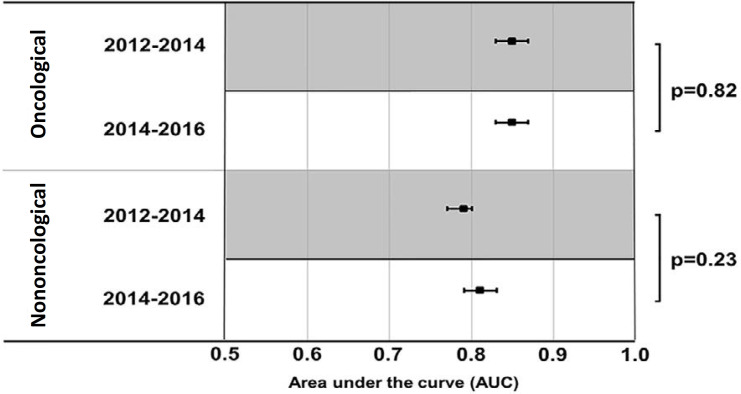
Area under the Receiver Operating Characteristic curves for Simplified Acute Physiology Score 3 in oncological (upper part) and nononcological patients (lower part). Subgroups were divided by the time period of intensive care unit admission (first and second half).

**Figure 3:**
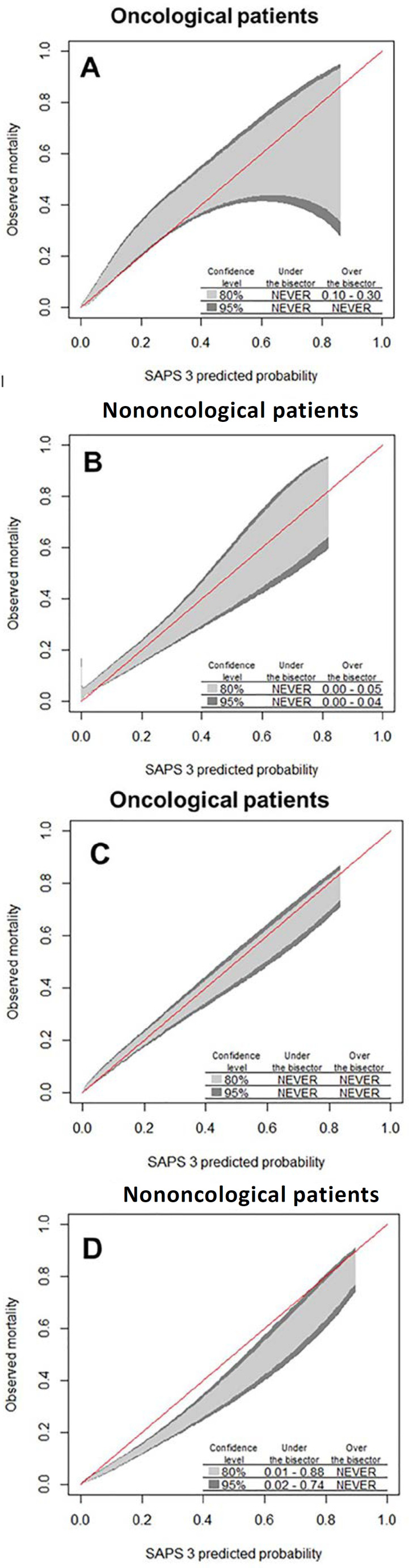
Calibration belt for Simplified Acute Physiology Score 3 in oncological (A and C) and nononcological patients (B and D), described as bisector deviation intervals. The predicted mortality intervals at which the calibration belt significantly deviates from the bisector and the 80% and 95% confidence levels are described in the lower right region of the plots. Subgroups were divided by the time period of intensive care unit admission (first period A and B; second period C and D). SAPS 3 - Simplified Acute Physiology Score 3.

The frequency of oncological patients did not change between the two time periods evaluated (1,498 of 3,542 patients [42.3%] *versus* 1,510 of 3,712 patients [40.7%], respectively; p = 0.16). The discrimination of SAPS 3 was not affected within subgroups by time period (Figure 2). Calibration belt analysis showed no miscalibration in the oncological subgroup of patients within either period. However, in the nononcological group, undercalibration was observed in the first period, and overcalibration was observed in the second period (Figure 3 and Table 3).

**Table 3:** SAPS 3 performance for oncological versus nononcological patients in the first and second periods

	SMR (95%CI)	Discrimination AUROC (95%CI)	Calibration*		Precision
			Over the bisector 95%CI	Under the bisector 95%CI	Brier score
First period (2012 - 2014)					
Oncological patients	1.27 (1.15 - 1.40)	0.85 (0.83 - 0.87)	Never	Never	0.10
Nononcological patients	1.08 (0.95 - 1.20)	0.79 (0.77 - 0.80)	0.00 - 0.04	Never	0.09
Second period (2014 - 2016)					
Oncological patients	1.01 (0.91 - 1.12)	0.85 (0.83 - 0.87)	Never	Never	0.095
Nononcological patients	0.71 (0.62 - 0.81)	0.81 (0.79 - 0.83)	Never	0.02 - 0.74	0.075

SMR - standardized mortality rate; 95%CI - 95% confidence interval; AUROC - area under the Receiver Operating Characteristic curve.

* Calibration described as the bisector deviation intervals using the calibration belt method.

## DISCUSSION

In this retrospective Brazilian cohort, we evaluated SAPS 3 performance in both oncological and nononcological critically ill patients. We found that (1) the SAPS 3 discrimination and calibration were accurate in our cohort for oncological patients; (2) the discrimination was greater and calibration was more accurate for oncological patients than for nononcological patients; and (3) the calibration was affected by the time period only for nononcological patients.

Recently, Moralez et al. published the largest validation study of severity-of-illness scores in Brazil using contemporary data from a multicenter cohort.^(6)^ They showed that the SAPS 3 standard equation was accurate in predicting outcomes in our country, supporting the national initiative from the *Associação de Medicina Intensiva Brasileira* (AMIB) for benchmarking units.^(18)^ However, the case-mix variation between units may lead to performance deterioration.^(19)^ A particular subgroup of interest is critically ill oncological patients because 15% of the patients admitted to European ICUs have cancer.^(20)^

Some previous publications evaluated SAPS 3 performance in oncological patients.^(8-10)^ Overall, these studies suggest that the measure is accurate for both discrimination and calibration, as demonstrated by our data. This finding is reassuring because oncological patients are usually poorly represented in development cohorts (8% in the original SAPS 3 cohort).^(4,5)^ Nevertheless, the hospital mortality of critically ill patients with cancer depends on acuity rather than on the presence and characteristics of the malignant disease itself.^(20,21)^ Therefore, the general severity of illness scores might capture most of the short-term prognosis in this population.

A novel approach of the present study was the comparison of SAPS 3 performance in oncological *versus* nononcological patients. We observed that this score was superior in oncological patients admitted to the ICU in terms of discrimination and calibration. To the best of our knowledge, this is the first study to compare these subgroups in the same cohort. The reasons for this observation are unclear but highlight the importance of case-mix differences between units and how this might affect model comparisons.

Another relevant observation was the effect of time on performance components. Calibration is particularly susceptible to time trends. Zimmerman et al. showed that the discrimination of Acute Physiology And Chronic Health Evaluation (APACHE) IV was robust and changed little throughout the evaluated time period but that calibration deteriorated in the general ICU population.^(11)^ Soares et al. also demonstrated this deterioration in their temporal analysis of SAPS 3 calibration in oncological patients.^(8)^ Again, we observed different effects of the time trend analysis in oncological compared with nononcological patients. No miscalibration was observed in oncological patients in either period. However, not only did miscalibration occur in nononcological patients but it also moved from underestimation to overestimation from the first to the latest period evaluated. We speculated that clinical practice changes affected mainly nononcological patients, but this suggestion may be oversimplistic. Selection bias at ICU preadmission and end-of-life practice may also affect calibration.

Our study has some strengths, such as a large sample size, which allowed the performance of hypothesis-generating subgroup analyses (oncological *versus* nononcological patients) not previously performed. Our study also evaluated a score validated in the same settings as in previous studies in oncological patients as well as in one of the largest external validation cohorts.^(6)^ However, this study also has limitations. First, it is a single-center cohort; our results reflect local practice and our case mix (which limits the generalizability of our study). Nevertheless, some of our results agree with previous study findings. Second, the study had a long period of data collection. Although this factor might affect the overall performance evaluation, it was required for the time period analysis. Finally, end-of-life decisions were not systematically annotated in our database.

## CONCLUSION

We observed that SAPS 3 discrimination was better for oncological than for nononcological patients and that SAPS3 showed no relevant deviations from optimal calibration in oncological patients. SAPS 3 performance (mainly calibration) varied over time differently according to the oncological status in our single-center cohort.
